# Supplementary material for: GTN Enhances Antitumor Effects of Doxorubicin in TNBC by Targeting the Immunosuppressive Activity of PMN-MDSC
Source: Cancers (Basel). 2023 Jun 9;15(12):3129. doi: 10.3390/cancers15123129 (PMC10296121; doi:10.3390/cancers15123129)
Supplement: Supplementary file 1 [file cancers-15-03129-s001.zip › cancers-2413872-supplementary.pdf]

Table S1. Forward and reverse primers

Primers (mouse)	Forward	Reverse
cxcr2	CAGTTCAACCAGCCCTGACA	ATCTTTGAGGTAAACTTAATCCTGC
fatp2	AAAGTAGACGGAGTGTCGGC	TTGGAAGACCTGTGGTTCCC
inos	GACCCTAAGAGTCACCAAAATGG	ATGCAGCTTGTCCAGGGATT
ppia	TCCTGGCATCTTGTCCAT	TGCTGGTCTTGCCATTCCT
actine	GGCACCACACCTTCTACAATGAGC	CGACCAGAGGCATACAGGGACAG

Table S2. Antibodies used for flow cytometry analysis

MDSCs / CD8+ Staining		
Antibodies	Clone	Supplier
CD45-Pacific Blue	30-F11	Biolegend
CD11b-APC	M1/70	Biolegend
PD-L1-PE/ IgG2b-PE	10F.9G2/ eBR2a	Biolegend
Gr1-PE/Cy7	RB6-8C5	Biolegend
Ly6G-FITC	1A8	BD Pharmingen
CD8- PerCP/Cy5.5	53-5.8	Biolegend
PD-1-APC/ IgG2b-APC	J43/ eB149/10H5	eBiosciences
Fixable Viability Stain 700		BD Pharmingen

Supplementary Figure S1

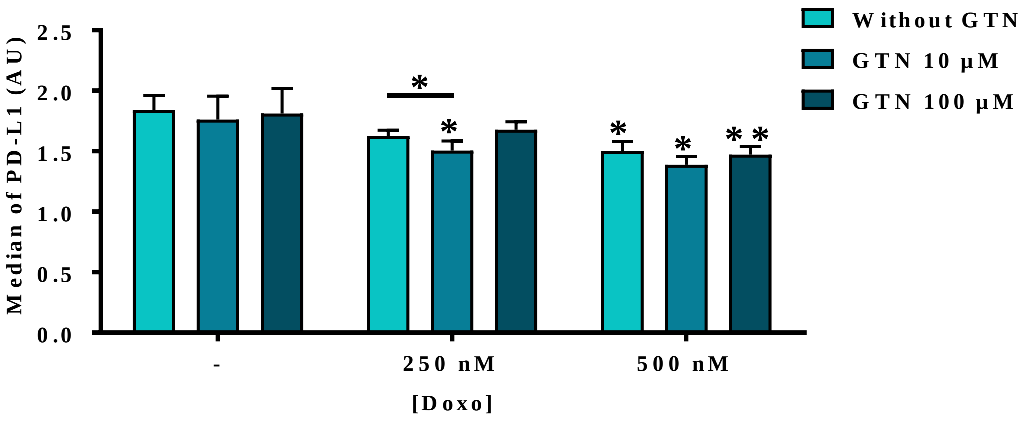

Figure S1. Modulation of PD-L1 expression in EMT6 breast mammary cells in response to doxorubicin +/- GTN. Five hundred thousand of EMT6 mammary cancer cells were treated with doxorubicin (250 or 500 nM) +/- GTN (10 or 100 µM) for 24 (n=4). The expression of PD-L1 was analyzed by flow cytometry. Statistical analysis were performed by t-test : * $p \leq 0.05$, ** $p \leq 0.01$. Isolated stars correspond to significant differences relative to Ctrl.

Supplementary Figure S2

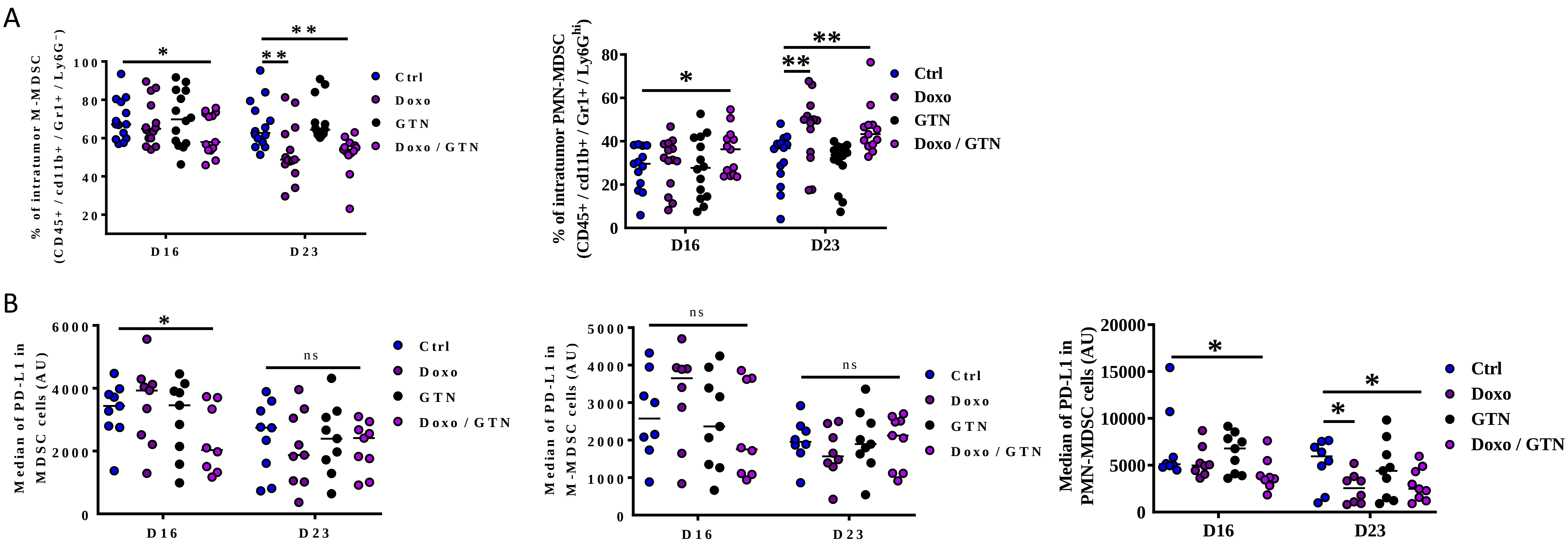

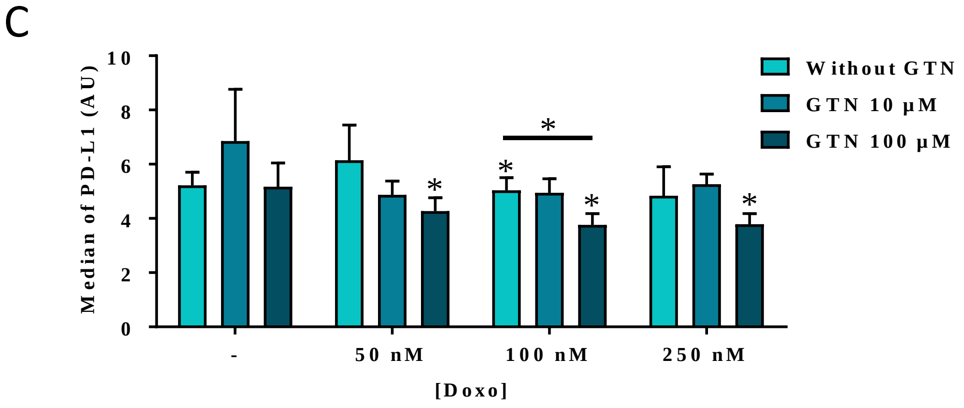

Figure S2. Modulation of MDSCs tumor infiltration and PD-L1 expression in response to doxorubicin +/- GTN. Flow cytometry analysis of MDSCs subtype tumor infiltration (A) or PD-L1 expression in tumor infiltrated MDSCs, M-MDSC, and PMN-MDSCs (B) or MSC2 cell line (C) in response to doxorubicin +/- GTN. Intratumor MDSC (A-B) were harvested on D16 and D23 post-injection of 4T1 cells into Balb/C mice (see Figure 1A) (n = 9). Five hundred thousand of MSC2 (C) were treated with doxorubicin (100 or 250 nM) +/- GTN (10 or 100 µM) for 48 hours (n=3). Statistical analyzes were performed by t test: * p ≤ 0.05, ** p ≤ 0.01, ns = not significant. Isolated stars correspond to significant differences relative to Ctrl.

Supplementary Figure S3

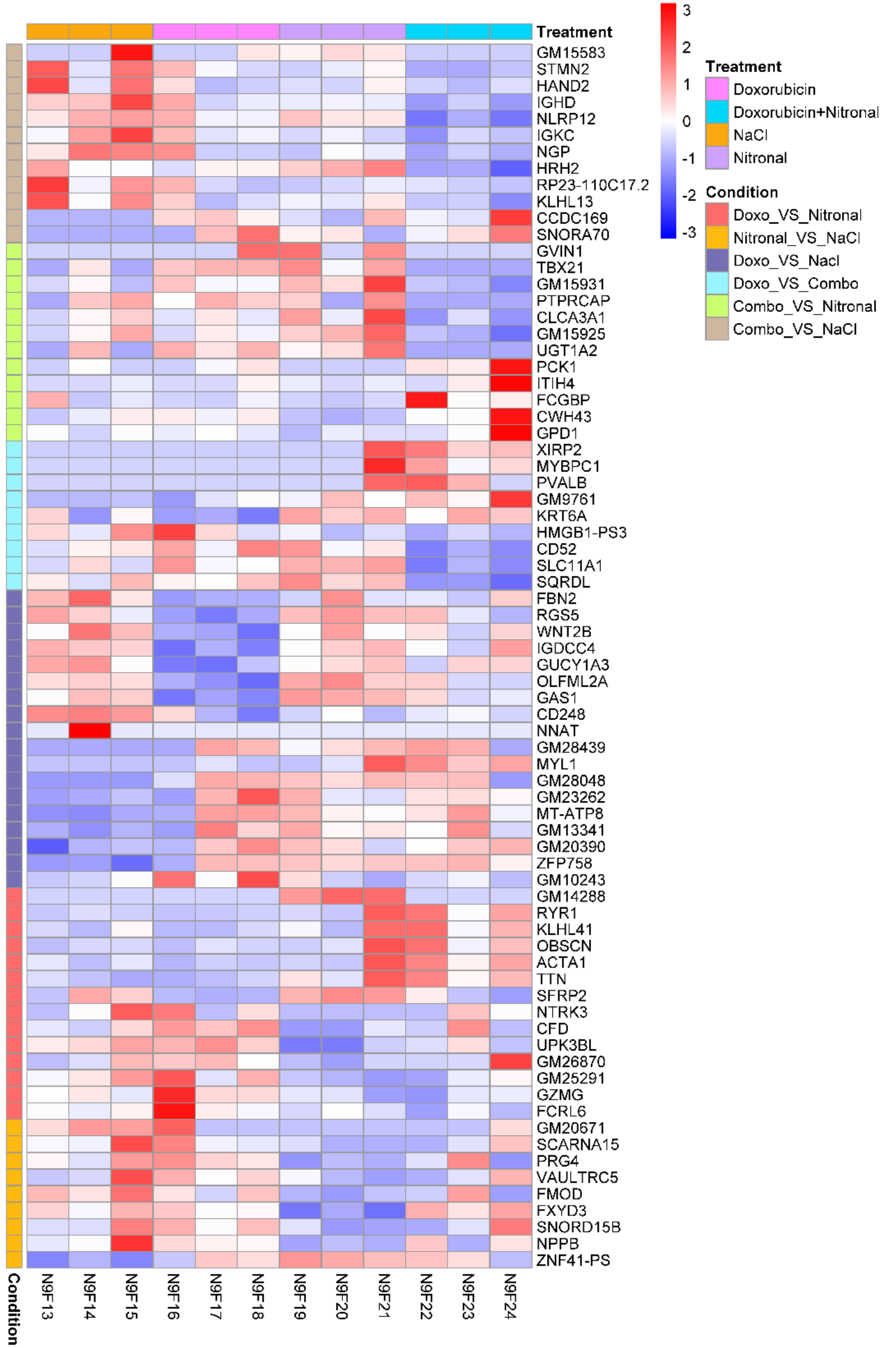

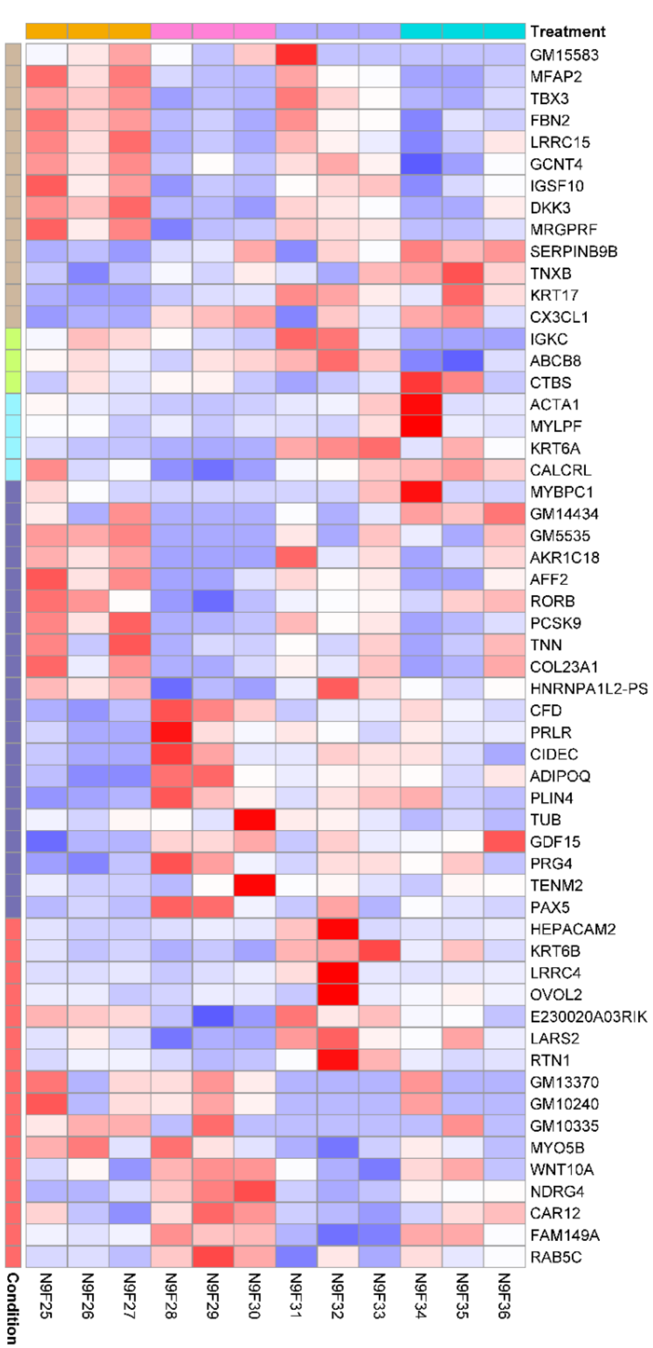

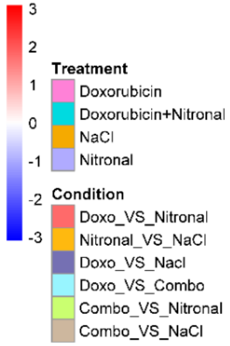

Figure S3. Expression heatmap for genes expression variation, in response to doxorubicin +/- GTN. RNAseq analysis of the intra-tumor expression of genes, in response to doxorubicin +/- GTN (Nitronal) on D16 (left) and D23 (right) post-injection of 4T1 cells into Balb/C mice (n = 3 mice) (see Figure 1A). Statistical analyzes were performed by the Wald test (DESeq2 R package).

Supplementary Figure S4

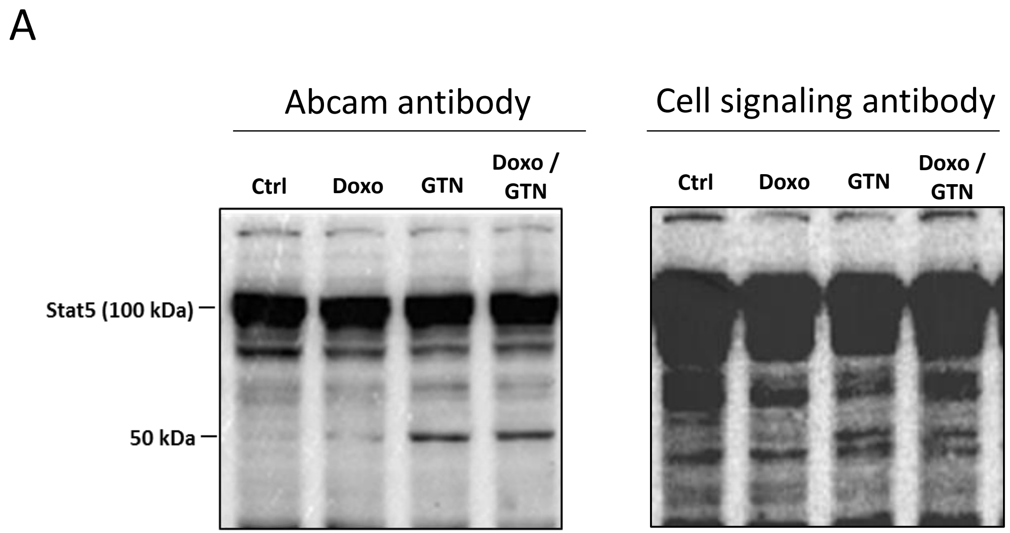

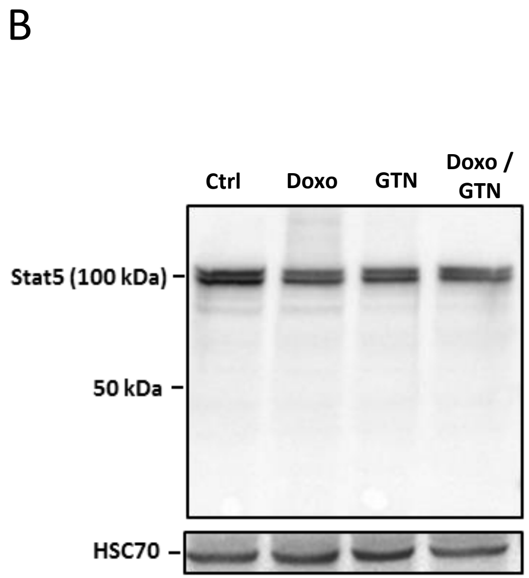

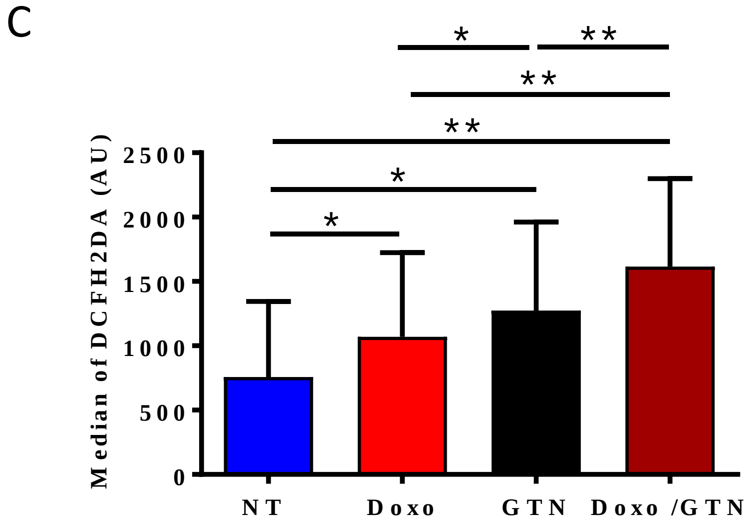

Figure S4. STAT5 cleavage in response to doxorubicin +/- GTN. A.B. Western blot analysis of STAT5 cleavage in MSC2 cells (A, n=3) or in CD8 TILs, purified from naive mice spleen and activated with anti-CD3/CD28 beads (B), treated with doxorubicin (100 nM) +/- GTN (100 µM) for 48h. STAT5 cleavage in MSC2 cells was detected by two different antibodies (ab), a polyclonal ab from Abcam (left) and a monoclonal ab from Cell signaling (right). **C.** Flow cytometry analysis of ROS production, analysed by DCFH2DA staining, in MSC2 cells treated with doxorubicin (100 nM) +/- GTN (100 µM) for 48h (n=4). Isolated stars correspond to significant differences relative to Ctrl. Statistical analyzes were performed by t test: * $p \leq 0.05$, ** $p \leq 0.01$, ns = not significant.

Supplementary Figure S5

A

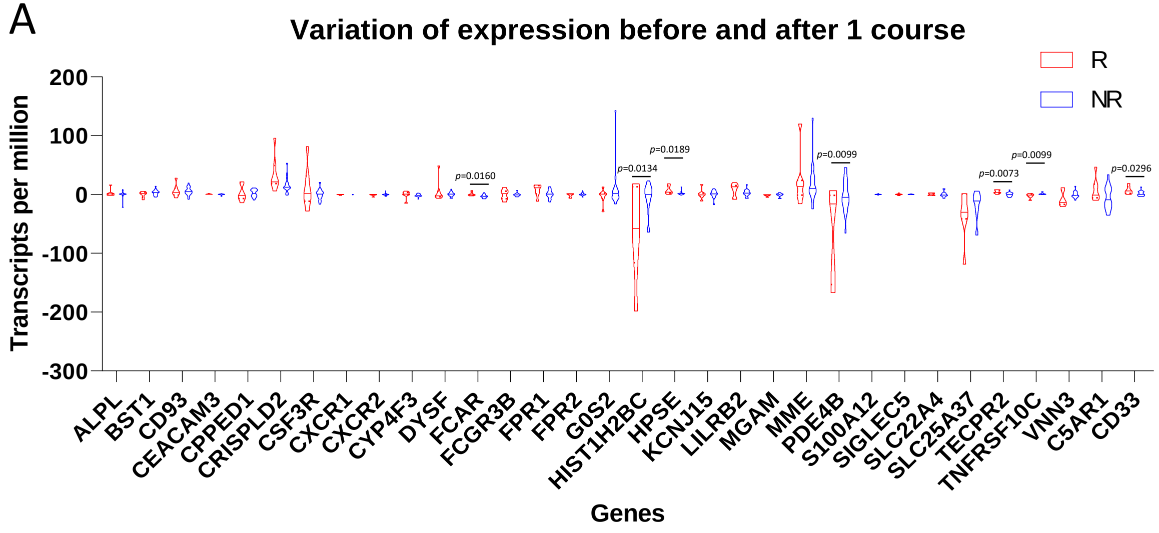

B

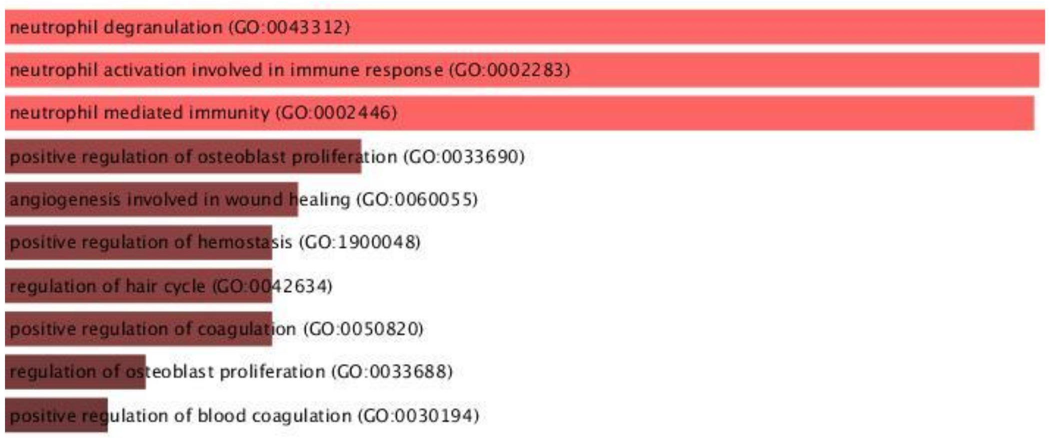

C

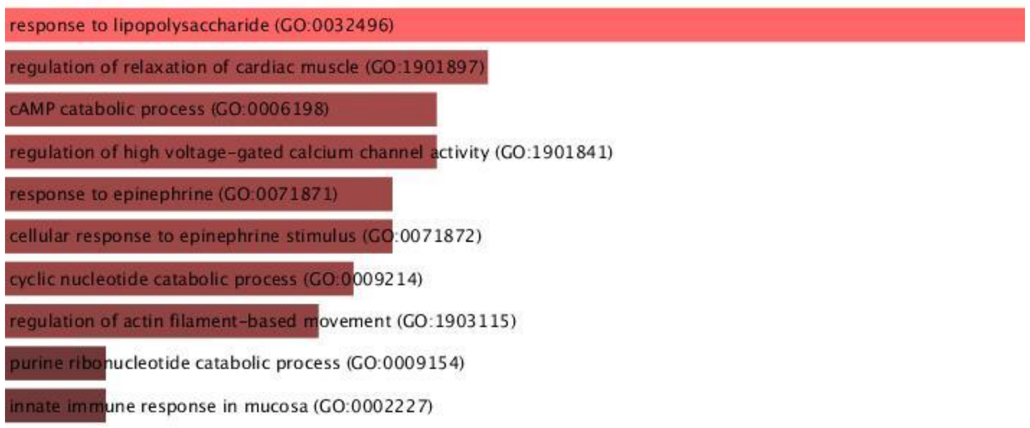

Figure S5. Variation of gene expression before and after the first dose of FEC/Docetaxel chemotherapy. A. RNAseq analysis of the intra-tumor expression of polymorphonuclear cells related genes, in good (red) and non responder (blue) breast cancer patients, before and after the first course of 5-Fluorouracil / Epirubicin / Cyclophosphamide / Docetaxel chemotherapy. Statistical analyzes were performed by t test. **B-C.** Functional gene networks up (B) or down (C) regulated obtained from Enrichr's web-based tool.
