# Supplementary material for: GTN Enhances Antitumor Effects of Doxorubicin in TNBC by Targeting the Immunosuppressive Activity of PMN-MDSC
Source: Cancers (Basel). 2023 Jun 9;15(12):3129. doi: 10.3390/cancers15123129 (PMC10296121; doi:10.3390/cancers15123129)
Supplement: Supplementary file 1 [file cancers-15-03129-s001.zip › cancers-2413872-Western blots-gels.pdf]

Gels figures 4 and 5

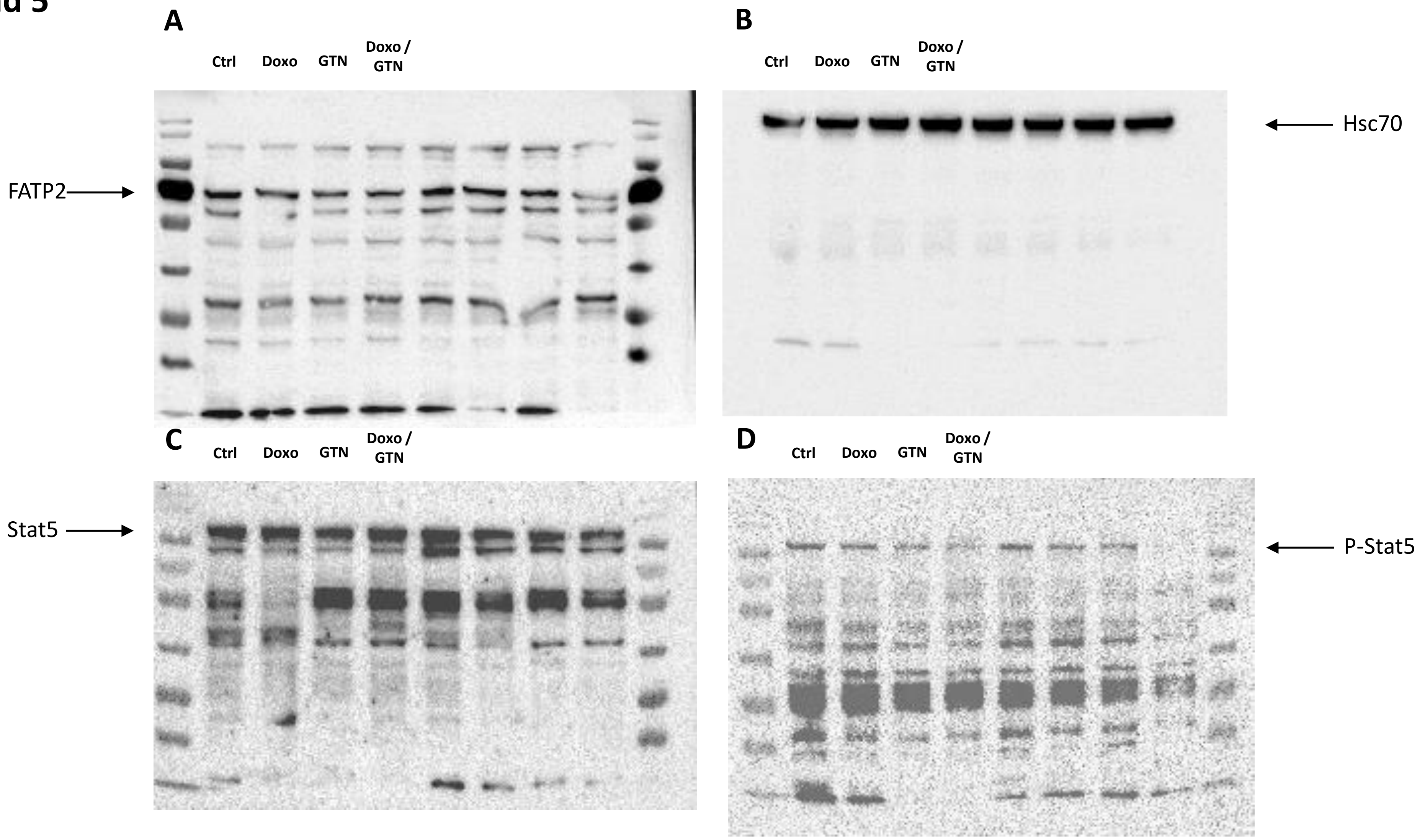

FATP2 and Stat5 expression in response to doxorubicin +/- GTN. A-D. Western blot analysis of FATP2 (A), Stat5 (C) or P-Stat5 (D) expression in MSC2 cells treated with doxorubicin (100 nM) +/- GTN (100 µM) for 48h. Detection of anti-Hsc70 antibody (B) served as a load control for western blot experiments.

Gels figures 5 and Sup figure S4

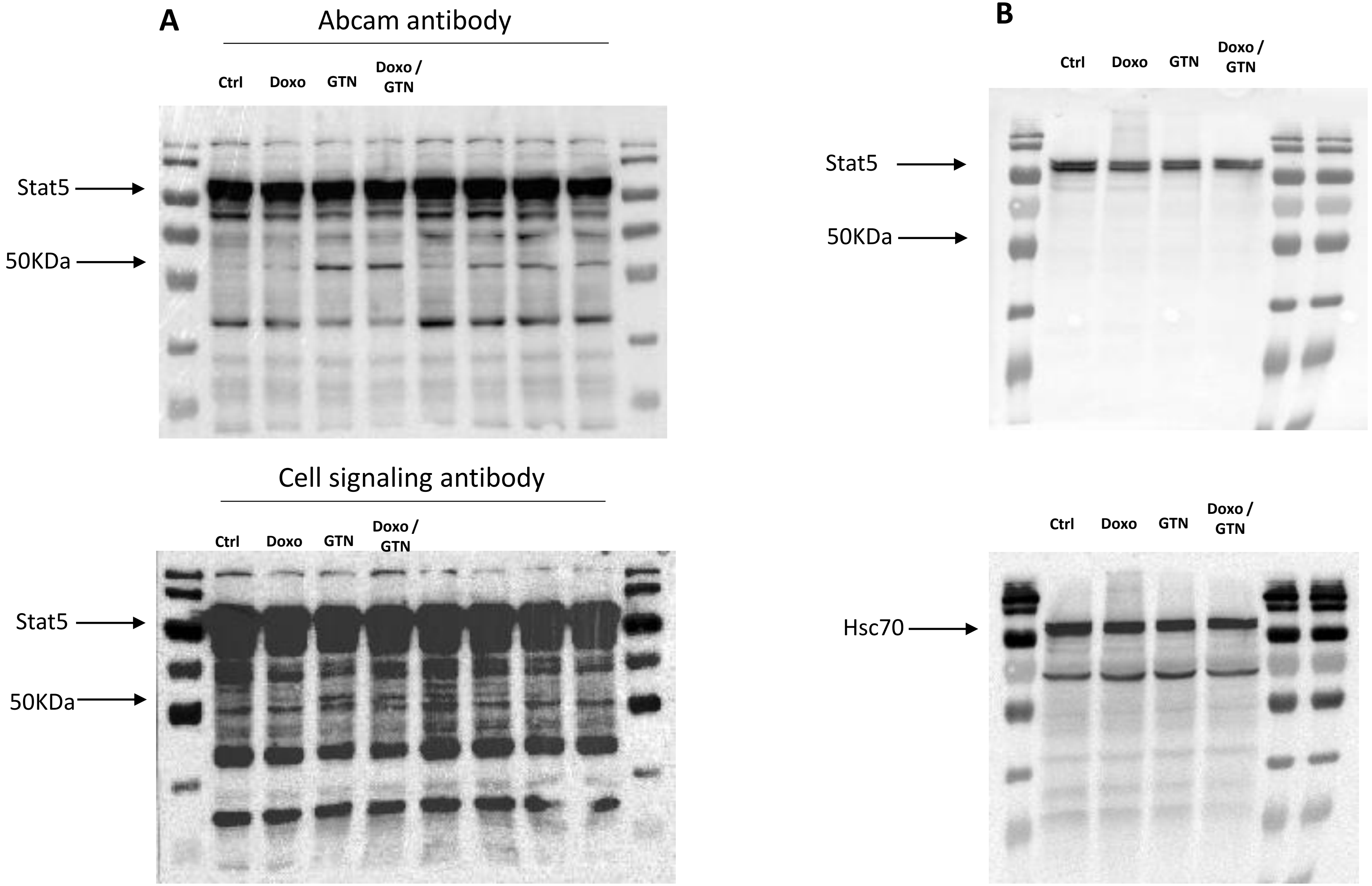

Stat5 cleavage in response to doxorubicin +/- GTN. A-B. Western blot analysis of STAT5 cleavage in MSC2 cells (A) or in CD8 TILs, purified from naive mice spleen and activated with anti-CD3/CD28 beads (B), treated with doxorubicin (100 nM) +/- GTN (100 µM) for 48h. STAT5 cleavage in MSC2 cells was detected by two different antibodies (ab), a polyclonal ab from Abcam (upper) and a monoclonal ab from Cell signaling lower). Detection of anti-Hsc70 antibody (B) served as a load control for western blot experiments.

Gels figure 5C

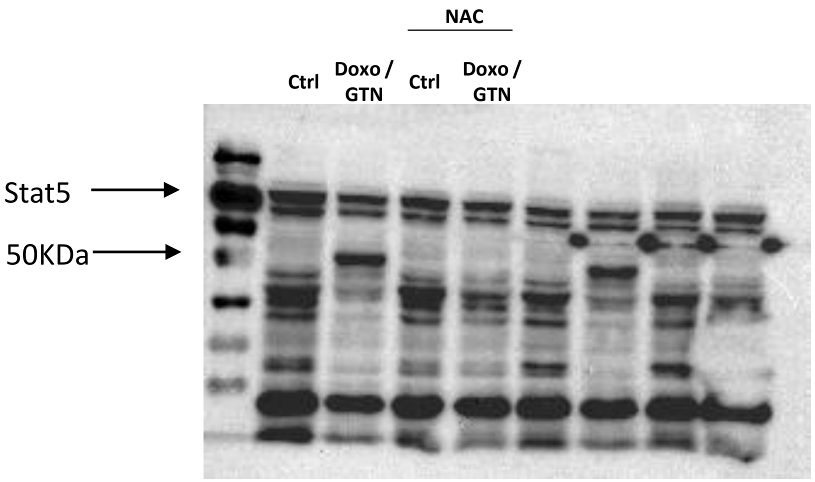

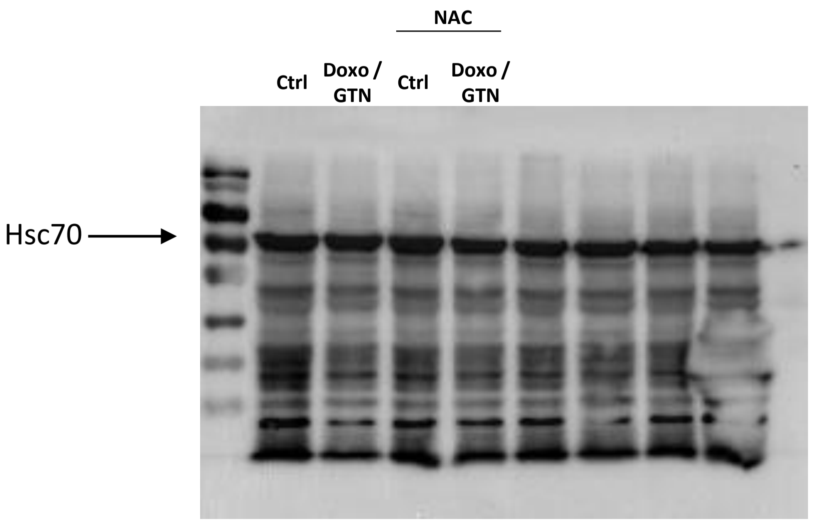

Involvement of ROS in FATP2 membrane expression. Western blot analysis of STAT5 cleavage in MSC2 cells treated with doxorubicin (100 nM) + GTN (100 µM) for 48h, with or without NAC (10 mM). Detection of anti-Hsc70 antibody served as a load control for western blot experiments. Isolated stars correspond to significant differences relative to Ctrl. Statistical analyzes were performed by t test: * $p \leq 0.05$, ** $p \leq 0.01$, ns = not significant.

Gels Figure 5D

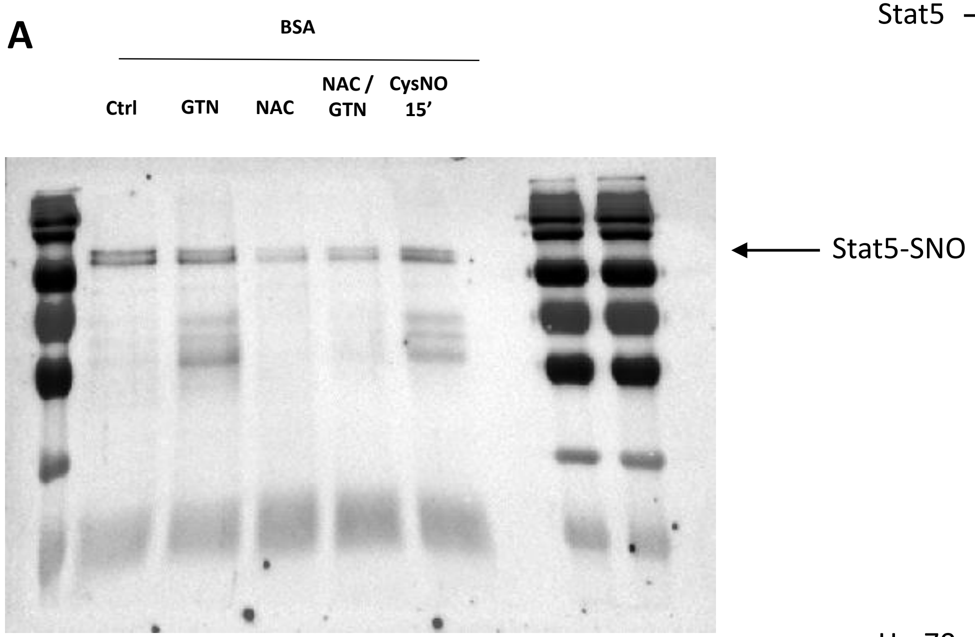

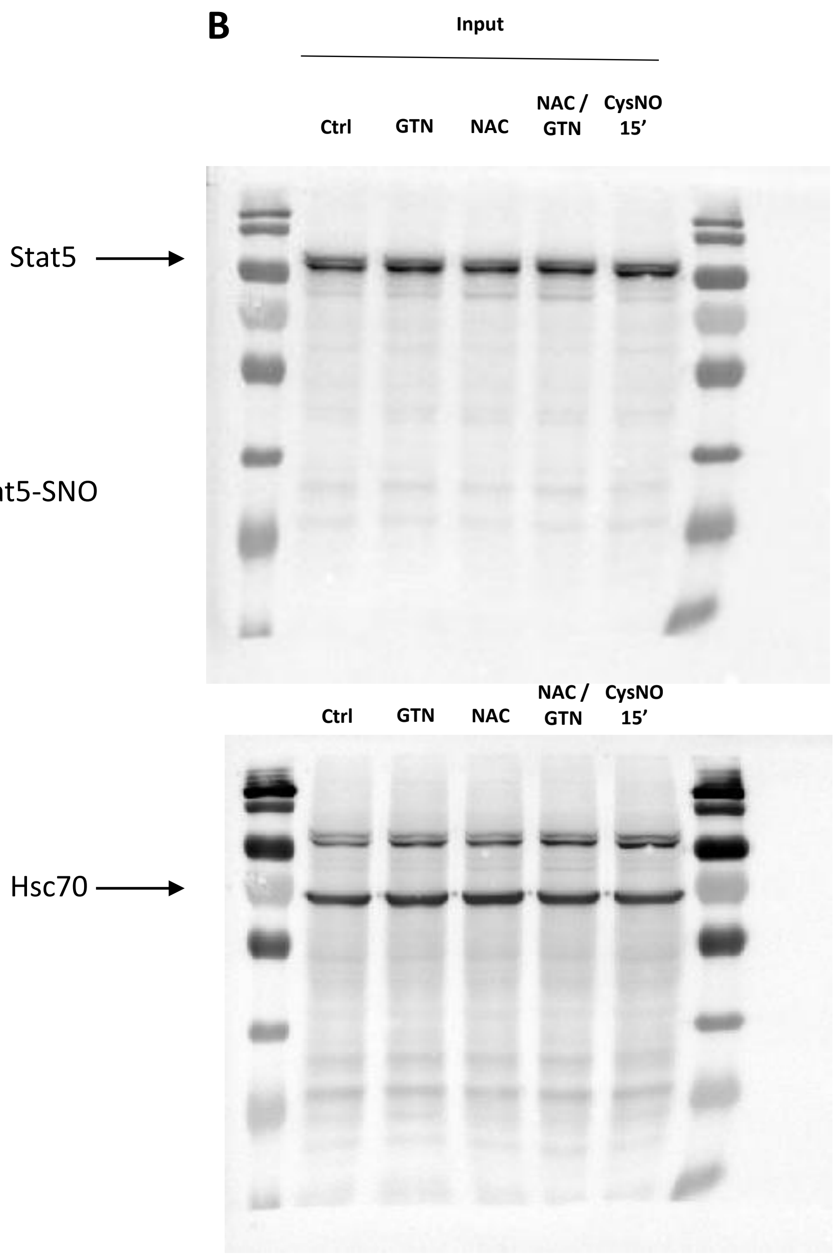

Figure S7: S-nitrosylation of Stat5. A-B. Western blot analysis of STAT5 expression in MSC2 cells treated with GTN (100 µM) for 48h, with or without NAC (10 mM). S-nitrosylation of Stat5 (Stat5-SNO) was detected after BSA (A). Detection of Stat5 and Hsc70 before BSA (B) served as a load control for western blot experiments.
